# Analysis of the elaboration and proposal of a Brazilian intersectoral strategy for the prevention and care of childhood obesity

**DOI:** 10.1590/0102-311XEN117722

**Published:** 2023-10-13

**Authors:** Gisele Ane Bortolini, Tatiane Nunes Pereira, Ariene Silva do Carmo, Ana Maria Thomaz Maya Martins, Jéssica Pedroso da Silva, Sara Araújo da Silva, Paloma Abelin Saldanha Marinho, Ana Maria Cavalcante de Lima, Ana Maria Spaniol, Gaia Salvador Claumann, Jonas Augusto Cardoso da Silveira, Juliana Rezende Melo da Silva

**Affiliations:** 1 Ministério do Desenvolvimento e Assistência Social, Família e Combate à Fome, Brasília, Brasil.; 2 Ministério da Saúde, Brasília, Brasil.; 3 Universidade Federal do Paraná, Curitiba, Brasil.

**Keywords:** Food Policy, Obesity, Child, Adolescent, Public Policy, Política de Alimentos, Obesidade, Criança, Adolescente, Política Pública, Política Alimentaria, Obesidad, Niño, Adolescente, Política Pública

## Abstract

The Brazilian Strategy for the Prevention and Care of Childhood Obesity (PROTEJA) aims to implement a set of actions to prevent obesity in Brazil. As such, this qualitative and descriptive documentary study presents the Strategy’s stages of the operational design, general proposal, evaluation and monitoring conducted by the Brazilian Ministry of Health’s technical coordination. After analyzing the epidemiological data on children and the existing policies aimed at obesity prevention, and reviewing the scientific literature and recommendations, PROTEJA was formulated and approved by the Brazilian Ministry of Health, and 1,320 municipalities committed to implementing 20 essential and 5 complementary actions, from 41, including some structural to improve environments. Coordinated by the Brazilian Ministry of Health in partnership with subnational governments and universities, the Strategy also relies on a local team for implementation support, as well as implementation and impact evaluations. Actions will be monitored annually, and the indicators will impact financial incentives. As a strong, evidence-based and innovative strategy aiming to promote healthy environments in cities, PROTEJA has the potential to open a path to childhood obesity reversal, as well as add to the implementation science and contribute to the development and improvement of public policies for obesity prevention; however, its implementation remains a challenge.

## Resumo

A Estratégia de Prevenção e Atenção à Obesidade Infantil (PROTEJA) visa promover a implementação de um pacote de ações para prevenção da obesidade no Brasil. Este estudo apresenta as etapas de desenho operacional, proposta geral, avaliação e monitoramento do PROTEJA realizadas pela coordenação técnica do Ministério da Saúde. Trata-se de um estudo qualitativo baseado em análise descritiva documental. A formulação da estratégia envolveu análise de dados epidemiológicos de crianças brasileiras, análise de políticas existentes, e uma revisão da literatura científica e das recomendações O PROTEJA foi então formulado e aprovado pelo Ministério da Saúde e 1.320 municípios se comprometeram a implementar 20 ações essenciais e 5 complementares das 41 ações, incluindo algumas estruturais para melhoria dos ambientes. A estratégia é coordenada pelo Ministério da Saúde em parceria com governos subnacionais e universidades. Sua implementação conta com apoio de uma equipe em nível local, e prevê a avaliação da implementação e de impacto. As ações serão monitoradas anualmente, e os indicadores também implicarão nos incentivos financeiros. O PROTEJA é uma estratégia forte, baseada em evidências e inovadora que visa promover ambientes saudáveis nas cidades, mas sua implementação é desafiadora. Não obstante, apresenta um possível caminho para a reversão da obesidade infantil. Também pode agregar à ciência de implementação e contribuir para o desenvolvimento e aprimoramento de políticas públicas de prevenção da obesidade.

## Resumen

La Estrategia Brasileña para la Prevención y Atención de la Obesidad Infantil (PROTEJA) tiene como objetivo promover la implementación de un conjunto de acciones para prevenir la obesidad en Brasil. Este estudio presenta las etapas del diseño operativo, la propuesta general, la evaluación y el seguimiento de PROTEJA realizadas por la coordinación técnica del Ministerio de Salud brasileño. Se trata de un estudio cualitativo basado en el análisis descriptivo de documentos. La formulación de la estrategia implicó el análisis de datos epidemiológicos de niños brasileños, el análisis de las políticas existentes y una revisión de la literatura científica y de las recomendaciones. PROTEJA fue entonces formulado y aprobado por el Ministerio de Salud, y 1.320 municipios se comprometieron a implementar 20 acciones esenciales y 5 complementarias de las 41 acciones, incluidas algunas estructurales para mejorar los entornos. La estrategia es coordinada por el Ministerio de Salud en colaboración con gobiernos subnacionales y universidades. Su implementación cuenta con el apoyo de un equipo a nivel local, y prevé una evaluación de la implementación y de impacto. Las acciones se monitorearán anualmente, y los indicadores también implicarán incentivos económicos. PROTEJA es una estrategia sólida, basada en evidencias e innovadora que busca promover entornos saludables en las ciudades, pero su implementación es un desafío. Sin embargo, presenta una posible vía para revertir la obesidad infantil. También puede agregar a la ciencia de implementación y contribuir al desarrollo y a la mejora de políticas públicas para prevenir la obesidad.

## Introduction

Childhood obesity in Latin America and the Caribbean is increasing significantly, resulting in a series of negative health repercussions in the short, medium, and long term and in overloaded health systems, generating high costs related to its treatment and complications in all age groups ^1^ ^,^ ^2^ ^,^ ^3^ ^,^ ^4^. This scenario is concomitant to undernutrition and the consequences of climate change, which are related, among other factors, to the population’s worsening eating habits, and to the increasing sales of ultra-processed foods that replaced traditional diets ^5^.

Given its complex and multifactorial etiology, preventing and controlling childhood obesity requires integrated actions ranging from individual to collective and environmental measures ^6^ ^,^ ^7^ ^,^ ^8^ ^,^ ^9^. Decision makers must recognize that childhood obesity is determined by individual, behavioral, and environmental factors - including family, community, school, social, and political contexts ^10^ ^,^ ^11^ -; that dietary practices determine population health and nutrition; that inadequate eating habits are a risk factor for malnutrition in all its forms, including overweight and obesity ^12^; and that physical inactivity and excessive sedentary behavior are among the main risk factors for overweight and obesity development and worsening in children ^10^ ^,^ ^11^.

When considering the social, economic, and political factors involved in determining obesity, it is essential we include the remodeling of municipalities as spaces that promote health as a transversal and priority theme on the public agenda. Thus, despite the leadership role assumed by the health sector in promoting these actions, other sectors are fundamental to support healthy eating and exercise, the main direct determinants of obesity ^6^ ^,^ ^7^ ^,^ ^8^ ^,^ ^9^.

From 2014 to 2019, Latin American and Caribbean countries committed to implementing the Pan American Health Organization’s (PAHO) Plan of Action for the Prevention of Obesity in Children and Adolescents, which aimed to fight against the rising epidemic of obesity in children and adolescents ^13^ ^,^ ^14^. Aligned to PAHO’s Plan, a World Health Organization (WHO) Commission on Ending Obesity published a report ^6^ with recommended actions to promote healthy eating habits and exercise, actions related to preconception and pregnancy care, early childhood diet and exercise, health, nutrition and exercise for school-age children, and weight management ^14^. Moreover, several international institutions analyzed and published best practices to prevent obesity ^3^ ^,^ ^7^ and to promote healthier cities ^15^. All documents and current scientific evidence pointed to the need to establish a multisectoral policy with well-structured governance to address the issue.

Over the years, the Brazilian government has participated in discussions on the topic and identified the importance of elaborating a multisectoral strategy integrating the recommendations to create healthy municipalities to combat childhood obesity. The proposed public policy, presented by Brazil in 2021 and considered innovative by the WHO and the United Nations (UN) ^16^, named Strategy for the Prevention and Care of Childhood Obesity (PROTEJA; *Estratégia de Prevenção e Atenção à Obesidade Infantil*), is an intersectoral strategy aiming to stop the advance of childhood obesity. Thus, in order to share the Brazilian experience with national and international researchers and decision-makers, this paper presents the Strategy’s stages of operational design, general proposal, evaluation and monitoring conducted by the Brazilian Ministry of Health’s technical coordination.

## Methods

This qualitative documentary study analyzed documents published between 2017 and 2022, such as standards, technical materials and accountability public reports produced annually by the Brazilian Ministry of Health’s General Coordination of Food and Nutrition. Bibliographical searches were conducted on grey literature and official government websites due to the originality of our scope.

Information about the main aspects of the elaboration process and the partnerships established with Universities to support implementation and evaluation were collected from the aforementioned accountability public reports. The Brazilian Food and Nutrition Surveillance System and national surveys on the topic were important references for developing the Strategy.

Information about PROTEJA’s general goals, operation, monitoring and payment of the financial incentive were retrieved from public technical notes published by the Brazilian Ministry of Health.

To characterize the complementary actions selected by municipalities upon adherence to PROTEJA in 2021, we conducted a descriptive analysis using information from an official primary health care (PHC) system ^17^ and normative acts ^18^. We also calculated the distribution of relative and absolute frequencies.

Our results will be presented according to the phases of a public policy cycle: from formulation, which covers problem identification and characterization of intervention and an evaluation of possible solutions and its costs ^19^ ^,^ ^20^ ^,^ ^21^, to implementation and monitoring and evaluation strategies, based on evaluation and implementation processes theories ^21^ ^,^ ^22^.

## Results and discussion

Public policy elaboration follow certain phases that we could organize into formulation, presented between Steps 1 to 4; implementation, described in Steps 5 and 6; and lastly monitoring and evaluation, presented in Step 7.

### Step 1 - Recognizing the need to elaborate a national strategy to prevent childhood obesity

Any health public policy must be based on local epidemiological data and current scientific evidence ^23^. According to Kingdon ^19^, decision-makers use indicators to evaluate the problem’s magnitude and identify changes. In Brazil, policy making supporting data was ensured by the implementation of an expanded food and nutrition surveillance strategy that includes monitoring nutritional status and food consumption in PHC, as well as national surveys ^24^ ^,^ ^25^.

Data showed an increase in the prevalence of childhood overweight (which includes overweight and obesity) ^26^ ^,^ ^27^. Regarding children under 5 years old, time trend analysis of population surveys showed a 160% increase in the prevalence of overweight between 1989 (3%) and 2006 (7.8%) ^27^. A recent national survey showed that 10% of Brazilian children under 5 were overweight and 3% were obese in 2019 ^28^. In the 34 years between 1974-1975 and 2008-2009, data shows a significant increase in the prevalence of overweight, from 10.9% to 34.8% among boys and from 8.6% to 32% among girls aged 5-9 years ^27^. As for the nutritional status of children monitored in PHC in 2021, 15.8% of children under 5 years of age and 33.9% of children aged 5-9 years were overweight and, of these, 7.6% and 17.8%, respectively, were obese ^29^.

Among children under 6 months of age, 45.7% were exclusively breastfed ^27^. At 12 months, 53.1% of children were continuously breastfed and, among children under 24 months, 60.9% were breastfed the previous day, which represents an increase of 4.6 percentage points compared to 2006 data ^27^. Regarding children followed up in PHC in 2021, 53% of children under 6 months were exclusively breastfed ^29^. Among children aged 6-23 months, 67% had a diversified diet containing six food groups; on the other hand, 31% were fed at least one type of ultra-processed food the previous day. Children older than 2 years showed an even higher percentage, with 87.5% of children aged 2-4 years and 89% of children aged 5-9 years consuming at least one type of ultra-processed food the previous day ^30^.

As Brazil lacks a national survey designed specifically to measure children’s physical activity levels, we had to use the available scientific evidence on the topic. Results of a systematic review study noted variations in the prevalence of physical inactivity from 70.8% to 89.1% in children aged 6-10 years ^31^. Another systematic review showed that about 30% to 96.4% of children spend, respectively, more than two hours and more than four hours per day in sedentary activities such as using screens ^32^.

As seen, investments by the Brazilian government in national surveys were crucial to recognize the need to elaborate and implement a national strategy to prevent childhood obesity, as advocated by Brownson et al. ^23^.

### Step 2 - Analyzing the existing policies and their status

After recognizing the existence of a public health issue that needs to be solved based on epidemiological data, one must identify the policies that have already been implemented in the country and analyze their results to assess whether they should continue or undergo changes to achieve the new goals ^19^. This step enables integrating actions and policies around the same objective.

In fact, the Brazilian Ministry of Health has invested in a series of actions that contribute to preventing childhood obesity, such as food and nutritional surveillance, promotion of breastfeeding and complementary feeding, and promotion of healthy eating in schools ^33^ ^,^ ^34^ ^,^ ^35^, under the Brazilian Strategy for Breastfeeding and Complementary Promotion and the Brazilian Health at School Program (PSE, acronym in Portuguese) ^33^.

In addition to structured programs, the published dietary guidelines based on the level of food processing and sustainable principles have influenced important food programs, such as the Brazilian National School Feeding Program (PNAE, acronym in Portuguese), which serves public schools nationwide. These guidelines also underpin the work of PHC health professionals. Considering that the consumption of ultra-processed foods is associated with obesity prevalence in the country, implementing a guideline based on the NOVA classification can contribute to obesity prevention ^34^.

Despite the importance of these programs and actions, they are insufficient to stop and reverse the current scenario, as they only partially cover the actions recommended for the prevention and care of childhood obesity. Besides, they are implemented in an uncoordinated and isolated manner. This finding highlighted the need to move forward and coordinate and integrate more actions beyond the health and education sectors, to ensure a healthier childhood by creating healthy municipalities that promote healthy eating and exercise. The Federal Government ^36^ saw a successful attempt at intersectoral articulation for obesity prevention throughout the formulation of a national strategy for the prevention and control of obesity coordinated by a National Chamber of Food and Nutritional Security; however, both the strategy and the Chamber were discontinued in 2019.

### Step 3 - Reviewing the scientific literature and recommendations to prevent childhood obesity

An extensive review of the scientific literature and recommendations from international organizations is essential to identify alternatives being discussed by policy communities, verifying which have not been implemented to address the issue ^19^. Considering the complexity of childhood obesity and recognizing obesogenic environments as an important factor for obesity growth, a literature review was conducted to identify effective interventions, experiences from other countries and the best governance structures that Brazil could adopt. Moreover, recommendations from leading international health and food and nutrition security organizations, such as PAHO, WHO, Food and Agriculture Organization of the United Nations (FAO), United Nations Children’s Fund (UNICEF) and World Obesity, as well as other institutions, such as the World Bank and the Organisation for Economic Co-operation and Development (OECD), were also consulted.

To complement and validate the recommendations identified, the Brazilian Ministry of Health promoted a regional meeting on actions to prevent childhood obesity, within the framework of the United Nations Decade of Action for Nutrition, in which invited countries shared experiences and strategies to address childhood obesity ^37^; held a workshop with subnational references, experts and civil society organizations to discuss experiences, strategies, challenges and recommendations to advance the theme ^37^; commissioned three studies to map the scientific evidence on the food, nutrition and physical activity scenario and its determinants for children and adolescents, and developed a policy brief on childhood obesity to summarize the main evidence and recommended actions for local prevention and care of childhood obesity ^37^ ^,^ ^38^ ^,^ ^39^. These documents and discussions also included the participation of universities and international organizations, such as PAHO and the World Food Program ^37^.

In this regard, the Brazilian Ministry of Health identified that multicomponent interventions and intersectoral strategies should be adopted to prevent childhood obesity, focusing on its multiple determinants. Such measures should be adopted jointly to achieve a greater impact, i.e., they should not be implemented in isolation ^3^ ^,^ ^6^ ^,^ ^7^ ^,^ ^8^ ^,^ ^9^.

Moreover, the evidence points to the adoption of actions in different contexts, such as PHC, schools, healthy municipalities and related to communication and breastfeeding ^3^ ^,^ ^6^ ^,^ ^7^ ^,^ ^8^ ^,^ ^9^, as well as recommendations on governance ^40^ ^,^ ^41^ ^,^ ^42^ ^,^ ^43^ ^,^ ^44^ ^,^ ^45^ ^,^ ^46^ ^,^ ^47^ ^,^ ^48^ ^,^ ^49^. Box 1 describes the detailed actions.

**Box 1:** Recommendations identified in Step 3 (Reviewing the scientific literature and recommendations to prevent childhood obesity).

RECOMMENDATIONS ON ACTIONS	
Primary health care	Actions that allow to integrate different health services for obesity care, including strategies such as the training of health professionals; continuous monitoring of the nutritional status of children and families; actions to promote adequate and healthy food, breastfeeding and physical activity, and actions to prevent excessive weight gain, early diagnosis and adequate care for children, adolescents, and pregnant women
Schools	Protective measures that limit the promotion and availability of ultra-processed foods and sugary drinks, ensuring the availability of water and healthy foods. Educational actions with the school community, with strategies that promote the practice of physical activity, are recommended in these spaces
Healthy cities	Intersectoral actions and policies are needed to promote local food production and facilitate physical and financial access to healthy foods, strengthening family farming and agroecological production. It is also important to implement protective measures aimed at reducing exposure to unhealthy foods, and to create urban spaces for children to play and exercise
Public campaigns	Health communication campaigns that contribute to the dissemination of official guidelines for adequate and healthy eating and physical activity are recommended
Breastfeeding	Actions to protect, promote and support breastfeeding
RECOMMENDATIONS ON GOVERNANCE	
Strengthen national governance and ensure state leadership in solving the issue	
Create political will and increase political commitment and state responsibility toward the problem	
Reduce the influence of private interests in the definition of public policies and establish mechanisms to prevent and reduce conflicts of interest	
Expand social participation and transparency in the decision-making process	
Base the program on rights defined in the *Federal Constitution* as support to justify actions	
Monitor population data and partner with the academic sector, fund and use evidence in defining actions	
Act in an intersectoral manner, with adequate coordination and ensuring policy coherence	
Generate and guarantee sufficient and continuous resources (financial, personnel, infrastructure)	
Set measurable goals and independent monitoring	
Create accountability mechanisms for key policy stakeholders		

### Step 4 - Formulating the national strategy for the prevention and care of childhood obesity

This literature and recommendations review was the basis for developing a proposal for the Brazilian Strategy. Scientific evidence should underline a nutrition policy as this allows for greater capacity to advocate for chosen policies, countering arguments against their implementation and facilitating negotiations. Thus, partnerships between academics, policy makers and other advocates help to turn theoretical estimates of implementation into policy, map barriers and direct research to choose relevant objects for policy making and implementation ^45^ ^,^ ^46^.

After gathering all the recommendations, a Strategy proposal was drafted and preliminarily presented to groups of experts and organized civil society working with healthy eating and obesity, as well as to other sectors of federal and subnational decision-makers responsible for health and food and nutrition management, such as the Brazilian Ministry of Education and Citizenship. After collecting the suggestions, a new proposal was drafted and presented to an expert panel, who validated the proposal. As PROTEJA was launched within the Brazilian Unified National Health System (SUS), the Strategy was also validated by the Tripartite Commission of Interagencies.

In 2021, the Brazilian Ministry of Health launched PROTEJA, which guides municipalities to implement a set of actions that contribute to the health of children and adolescents. PROTEJA is a Brazilian intersectoral strategy that aims to stop the advance of childhood obesity and contribute to the care and improvement of childhood health and nutrition ^50^.

All states, the Federal District and municipalities can implement PROTEJA through activities in the lines described in Box 2 ^50^.

**Box 2:** Lines of activities to be implemented by the municipalities.

I	Food and nutrition surveillance, health promotion and prevention of excessive weight gain, early diagnosis and adequate care for children, adolescents and pregnant women within primary health care
II	Health promotion actions in schools to promote the consumption of adequate and healthy foods and the regular practice of physical activity
III	Education, communication, and information to promote healthy eating and physical activity for the Brazilian population
IV	Training and continuing education for professionals working in childcare
V	Intersectoral and community-based articulations that promote healthy environments and support healthy eating and the practice of physical activity in the cities

As this is an initiative led by the Brazilian Ministry of Health, including financial incentives, health managers at all government levels are primarily responsible for articulating with other sectors the implementation of this strategy in municipalities, such as education, social assistance, agriculture, food and nutrition security, urban development, sports, city halls, universities, civil society and organizations, among others. Nongovernmental actors without conflicts of interest and international agencies can also support childhood obesity prevention and care through their commitment to support the Strategy’s implementation in Brazil ^51^.

In the same year, the Brazilian Ministry of Health established a financial incentive to municipalities for implementing PROTEJA actions for the period 2021-2023 ^52^. Considering the available budget, the Brazilian Ministry of Health created prioritization criteria: municipalities with less than 30,000 inhabitants; prevalence of overweight greater than or equal to 15%; coverage of nutritional status assessment greater than or equal to 50%; and registration of food consumption markers assessment in health systems, the last three criteria referring to children under 10 years of age. Municipalities that met these criteria were invited and could agree or not to participate.

The financial resource transferred to municipalities for the implementation of PROTEJA actions will be distributed in three annual installments, from 2021 to 2023. Although financial resources are an important strategy to induce adherence and implementation, the available budget is considered only an incentive, as it is insufficient to implement all actions. However, management of the SUS, including its financing, is a common attribution to the Federal Government, and states and municipalities can complement the resources. Another limitation related to financing is that the financial transfer must be used to implement health services and actions, which is a challenge for intersectoral actions.

### Step 5 - Identifying the profile of agreed actions: adherence to the strategy

Among the eligible municipalities, 1,320 completed adherence (99.1%) and received federal financial resources for three years to support the local implementation of actions ^48^ ^,^ ^49^. In the adherence process, municipalities were presented with a list of 20 essential actions (Box 3) and 41 complementary actions (Table 1) for composing the strategy. All municipalities are committed to the 20 essential actions. From the 41 complementary actions, municipalities had to choose at least five actions.

**Box 3:** Essential actions recommended for the composition/reach of the Brazilian Strategy for the Prevention and Care of Childhood Obesity (PROTEJA) at the municipal level.

FIRST CONTACT (DIAGNOSIS AND CARE ACTIONS IN PHC)
Monitor the nutritional status and food consumption markers of children, adolescents and pregnant women according to the Brazilian Ministry of Health’s official documents Provide individual and collective multidisciplinary PHC care for pregnant women with pregestational excess weight or excessive gestational weight gain, according to the Brazilian Ministry of Health’s official documents Provide individual and collective multidisciplinary PHC care for children and adolescents diagnosed with overweight and obesity, according to the Brazilian Ministry of Health’s official documents Equip the UBS with at least one scale and stadiometer for adults and children, according to the Brazilian Ministry of Health’s regulations
ACCOUNTABILITY (COMMITMENT)
Elaborate the step-by-step plan for implementing PROTEJA
ORGANIZATION (MANAGEMENT)
Include, in the municipal health plan, goals for the prevention and care of childhood obesity agreed by the formal management and social control of the SUS, including representatives from other public management sectors Intersectoral articulation with the various related sectors for the local management of PROTEJA Include in the annual management reports the progress of the actions agreed by the municipality
TRANSFORMATION (FOOD AND NUTRITION EDUCATION AND STIMULUS OF PHYSICAL ACTIVITY)
Implement, strengthen and/or expand the EAAB in the municipality Carry out individual and collective actions of food and nutrition education and physical activity in UBS and other public spaces for children, adolescents and pregnant women Carry out food and nutrition education and physical activity actions in schools, mainly through the PHC Ensure at least 15 minutes of physical activity per day, in addition to curricular physical education classes, in all schools and at all levels of education
EDUCATION (TRAINING)
Qualify education and PHC professionals, including community health and social workers, on childhood obesity, based on the Brazilian Ministry of Health’s manuals, guides, and protocols
WINDOW OF OPPORTUNITY (COMMUNICATION)
Carry out institutional campaigns in the mass media on childhood obesity Make available PROTEJA’s printed and digital materials and the Brazilian Ministry of Health’s official guidelines on adequate and healthy food and physical activity in UBSs, CRAS, CAPS, Academy of Health centers, hospitals, and schools
ENVIRONMENTS
Comply with the provisions of article 22 of *Resolution n. 06/2020* of the FNDE on the PNAE Ensure healthy school canteens Create circuits of fairs and other healthy food marketing strategies that serve all regions of the municipality, especially in more vulnerable territories Promote and support urban agriculture, vegetable gardens in institutional settings, such as schools and health services, and in community spaces Map and qualify existing spaces and, if necessary, create new spaces for physical activity

CAPS: Centers for Psychosocial Care; CRAS: Social Assistance Reference Center; EAAB: Brazilian Strategy for Breastfeeding and Complementary Promotion; FNDE: Brazilian National Fund for the Development of Education; PHC: primary health care; PNAE: Brazilian National School Feeding Program; UBS: basic health units; SUS: Brazilian Unified National Health System.

**Table 1:** Complementary actions recommended for the composition/reach of the Brazilian Strategy for the Prevention and Care of Childhood Obesity (PROTEJA) at the municipal level.

Actions	n	%
Actions within PHC		
Manage excessive weight gain, gestational diabetes, and pregnancy-induced hypertension	851	64.5
Qualify the monitoring of physical activity actions	709	53.7
Organize care for childhood obesity, making other points in the health care network available for referral of severe cases of obesity	592	44.8
Provide at least one integrative and complementary practice option as part of the prevention and care of childhood obesity in PHC	544	41.2
Training actions (continuing education)		
Make available a workload so that PHC professionals in the municipality who work in the prevention of childhood obesity can attend at least one training or course per year, offered by the Brazilian Ministry of Health on the subject	854	64.7
Provide at least one training per year to PHC professionals who work, mainly with the maternal and child population, in anthropometric collection and evaluation of food consumption markers	821	62.2
Offer at least one training per year on healthy eating, physical activity and obesity for media professionals (journalists, advertisers, designers, etc.)	300	22.7
Establish partnerships with universities and colleges that have health courses to conduct research/outreach projects on the theme of childhood obesity in the municipality	181	13.7
Actions in schools		
Guarantee the free supply of drinking water (water fountains in adequate sanitary conditions) in public schools	774	58.6
Carry out the minimum purchase of family farming products for the PNAE with Federal Government resources, in accordance with current legislation, creating mechanisms to gradually increase the percentage of purchases in partnership with local producers and other municipalities	440	33.3
Establish guidelines for the Municipal Health and Education Departments to provide food and nutrition education activities, based on the principles and guidelines of the Brazilian Ministry of Health’s food guides, the NBCAL, and the regulations of the FNDE	315	23.9
Invest in the construction and maintenance of school infrastructure for physical activity	258	19.5
Create local legislation to prevent the association of unhealthy foods with food and nutrition education activities, textbooks and sponsored events in schools	170	12.9
Install bike racks in schools and lockers to store school supplies	104	7.9
Comprehensive measures for health promotion and prevention of childhood obesity		
Hold a public hearing together with the Legislative Branch to discuss childhood obesity prevention	202	15.3
Present bills on structuring measures that promote environments conducive to appropriate and healthy habits and behaviors, and prevention of childhood obesity	124	9.4
Municipalities that promote healthy eating		
Publicize the support network for the promotion of adequate and healthy eating and physical activity in the municipality	471	35.7
Stimulate production chains that promote adequate and healthy eating, considering the stages of storage, supply and/or distribution of fruits and vegetables, which follow good agricultural practices and integrated production systems, valuing local food culture	172	13.0
Create contact networks between local producers and traders to stimulate the circulation and sale of fresh and minimally processed foods in the municipality	150	11.4
Train local traders and retailers in strategies to transform the food retail trade into a healthier environment	144	10.9
Implement subsidies for the production of fresh and minimally processed foods	128	9.7
Establish rules on the receipt of food donations in public establishments, either for on-site consumption or for distribution, promoting adequate and healthy food, health and dignity of the recipients of donations, in accordance with the principles of food guides for the Brazilian population	107	8.1
Create local legislation to make it mandatory to provide free filtered water in public spaces, such as parks and squares, and in restaurants, cafeterias, bars and similar establishments	98	7.4
Create and disseminate an application mapping places that sell fresh and minimally processed foods	81	6.1
Create local legislation to establish a minimum height of 120cm for shelves displaying ultra-processed foods to protect children from grabbing these products at points of sale	81	6.1
Actions to support and protect breastfeeding		
Promote actions to encourage and support breastfeeding in daycare centers and schools, aiming at breastfeeding continuity	654	49.5
Extend maternity leave to at least six months and paternity leave to at least 20 days for municipal employees	198	15.0
Implement municipal laws that guarantee the right of women to breastfeed in any space, whether public or private	198	15.0
Strengthen and improve the implementation and inspection of the NBCAL and its regulation (*Law n. 11,265/2006* and *Decree n. 9,579/2018*)	119	9.0
Implement breastfeeding support rooms for breastfeeding working women in municipal public offices and encourage their implementation in workplaces in other governmental and private spheres, as well as in places with large circulation of informal breastfeeding working women	118	8.9
Encourage the private sector to join the Corporate Citizen program, pursuant to *Law n. 11,770/2008* and regulated by *Decree n. 7,052/2009*	89	6.7
Implement and/or expand the network of collection points and Human Milk Banks	80	6.1
Implement and/or strengthen and expand the IHAC in the municipal public health care network, as well as encourage this initiative in hospitals managed by other governmental and private spheres (*Ordinance n. 1,153/2014 - PRC n. 06/2017*)	74	5.6
Municipalities that promote physical activity		
Carry out regular leisure activities that involve physical activity in public places in cities	652	49.4
Invest in adapting and adjusting physical activity equipment for children with obesity	295	22.3
Prioritize areas of greater social vulnerability for investment in adequate structure for exercising	253	19.2
Create “leisure streets” (streets open to pedestrians) for at least one day a week for physical activity and sports	231	17.5
Implement programs and actions that provide adequate conditions for the active commuting of children and adolescents from home to school	204	15.5
Establish partnerships with clubs and other private establishments so that they can be used free of charge by the general public for practice physical activities and sports	167	12.7
Invest in the construction and maintenance of sidewalks and bike lanes, prioritizing areas of greater social vulnerability	156	11.8
Create and encourage the use of an online platform to identify and assess the quality of public, community and social spaces and facilities that can be used for physical activity	94	7.1

FNDE: Brazilian National Fund for the Development of Education; IHAC: Baby-Friendly Hospital Initiative; NBCAL: Brazilian Code of Marketing of Infant and Toddlers Food, Nipples, Pacifiers and Baby Bottles; PHC: primary health care; PNAE: Brazilian National School Feeding Program.

The most frequently agreed upon actions in the PHC context were “manage excessive weight gain, gestational diabetes and pregnancy-induced hypertension” (n = 851) and “qualify the monitoring of physical activity actions” (n = 709). The most selected actions related to training and continuing education were “make available a workload so that PHC professionals in the municipality who work in the prevention of childhood obesity can attend at least one training or course per year, offered by the Brazilian Ministry of Health on the subject” (n = 854) and “provide at least one training per year for PHC professionals who work, mainly with the maternal and child population, in anthropometric collection and evaluation of food consumption markers” (n = 821) (Table 1).

Regarding actions in schools, the following stand out: “guarantee the free supply of drinking water (water fountains in adequate sanitary conditions) in public schools” (n = 774) and “carry out the minimum purchase of family farming products for the PNAE with Federal Government resources, in accordance with current legislation, creating mechanisms to gradually increase the percentage of purchases in partnership with local producers and other municipalities” (n = 440) (Table 1).

As for the broad measures to promote health and prevent childhood obesity, the action “hold a public hearing together with the Legislative Branch to discuss childhood obesity prevention” (n = 202) stands out. Considering the actions related to municipalities that promote healthy eating, the most selected actions were “publicize the support network for the promotion of adequate and healthy eating and physical activity in the municipality” (n = 471) and “stimulate production chains that promote adequate and healthy eating, considering the stages of storage, supply and/or distribution of fruits and vegetables, which follow good agricultural practices and integrated production systems, valuing local food culture” (n = 172).

Regarding actions to support and protect breastfeeding, the most agreed upon actions were “promote actions to encourage and support breastfeeding in daycare centers and schools, aiming at breastfeeding continuity” (n = 654) and “extend maternity leave to at least 6 months and paternity leave to at least 20 days for municipal employees” (n = 198). As for actions that help municipalities promote physical activity, the most selected actions were “carry out regular leisure activities that involve physical activity in public places in cities” (n = 652) and “invest in adapting and adjusting physical activity equipment for children with obesity” (n = 295) (Table 1).

Although PROTEJA presents a list of innovative actions to be implemented by municipalities, most of them adhered to initiatives they were already developing, either in the health sector or in other sectors, and mainly related to childcare, rather than those addressing food environment and physical activity. Interventions to prevent obesity, which require greater state intervention to protect the health of the population, are more resistant to implementation by governments ^44^. Overcoming this barrier requires leadership from decision-makers, strong local governance and civil society support for these actions ^45^.

Moreover, intersectoral coordination of different sectors may be the first barrier to overcome in implementing PROTEJA, as identified in the analysis of some other national strategies. More recently, evaluation of a national program, whose objective was to address inequalities that protect socioeconomically vulnerable children, showed that the operationalization of its intersectoral actions was a barrier to effectiveness ^53^.

Considering the PROTEJA principles, which guarantee the universal and integral right to maternal and child health and the protection of children’s rights, as well as the social determinants and the interdisciplinary and intersectoral nature of the actions, the Strategy is expected to induce public managers and professionals from all sectors, civil society and partners to recognize childhood obesity as a priority public health issue and to share responsibility for implementing effective measures to prevent, care for and reverse childhood obesity in Brazil.

### Step 6 - Implementing PROTEJA: defining mechanisms to technically support municipalities

To stress the implementation process and increase its chance of success, many initiatives were foreseen in PROTEJA and it will be evaluated using implementation science in nutrition ^22^ to understand the barriers and facilitating factors of implementing nutrition programs in Brazil.

Governance of the PROTEJA implementation is based on institutional support, coordinated by the Brazilian Ministry of Health in partnership with subnational governments (state and municipal), considering that management of the SUS is inter-federative. Two guides were elaborated to support municipalities with systematic guidance on how to organize and implement each action.

To support the Brazilian Ministry of Health actions, a partnership was established with the Federal University of Alagoas (UFAL, acronym in Portuguese) to define a robust implementation process and independent monitoring and evaluation of the program ^37^. UFAL hired a team of four regional and 30 local supporters, who were trained in the PROTEJA themes and received supported from Brazilian Ministry of Health staff.

As support strategies, this team is in constant contact with states and municipalities and promotes virtual workshops for training, capacity building and support for implementing the Strategy’s actions for municipalities that have joined jointed PROTEJA. These workshops consider intersectoriality as the main pillar of PROTEJA and that actions aimed at changes in the food environment are rather difficult to implement. Thus, the first themes addressed aim to support the actors involved in local management on these themes, such as management and planning, discussing ways and possibilities to enable the meeting of different sectors to address the prevention and control of childhood obesity in municipalities.

To date, five workshops have been held in all states, with an average participation of 46.2% of municipalities. In total, 190 (14.4%) municipalities did not participate in any of these four workshops; 256 (19.4%) participated in only one; 284 (21.5%) participated in only two; 285 (21.6%) participated in only three; 209 (15.8%) participated in only four; and 96 (7.3%) participated in all five workshops held so far.

Specifically, to support implementation of the essential action “guarantee healthy canteens”, which all implicated municipalities must implement, and whose main objective is to approve regulatory standards in the municipalities, the Brazilian Ministry of Health hired the Federal University of Minas Gerais (UFMG, acronym in Portuguese). The support actions foreseen in this partnership are: conduct a situational diagnosis of the school food environment in PROTEJA municipalities; hold webinars to discuss the school food environment and support the implementation of the essential action “guarantee healthy school canteens”; monitor the implementation of actions related to the measures adopted to promote a healthy school food environment; elaborate a manual for healthy school canteens; and develop and offer a self-instructional training course for school canteen owners and municipal managers.

Moreover, the Brazilian Ministry of Health engaged with the State University of Rio de Janeiro (UERJ, acronym in Portuguese) to develop case studies in four PROTEJA municipalities in the referred state, mapping the challenges and opportunities to implementing actions that favor healthier environments and municipalities. This partnership will result in a guide for municipal managers to support planning and implementing policies that contribute to the building of environments favorable to health and the prevention of childhood obesity.

All workshops and support strategies are based on documents developed by the Brazilian Ministry of Health and universities. Besides these materials, published to support health professionals and PHC teams, the Federal Government launched an instructional course on the care of overweight and obese children and adolescents within the context of PHC ^37^.

Brazil is a Federation with more than 5,500 municipalities, which makes the implementation of any public policy a challenge. During the post-constitutional decentralization process, planning, evaluation and intersectoriality were already identified as problematic, and experts in federalism and social policies suggested enhancing local capacities to manage public policies ^54^ ^,^ ^55^.

### Step 7 - Monitoring and evaluation: making sure we are on the right track

To identify problems and failures capable of jeopardizing the actions, processes or objectives of public policies and thus gather information to correct directions or adjust implementation plans ^22^, the technical team established a monitoring strategy to be performed continuously. All PROTEJA actions will be monitored through official health systems (annual reports) and online questionnaires.

In all health systems, PHC monitors annually the indicators related to the number of children under 10 years of age with assessed nutritional status and food consumption and the individual follow-up of children with obesity. These indicators affect the financial incentives passed on to the municipalities, i.e., the resources will be transferred only to municipalities that have increased these numbers. Moreover, the UFAL developed online questionnaires to monitor the implementation details of each action. Partial and final monitoring is being carried out and will be presented to state and municipal managers.

The first monitoring of indicators showed that 1,292 municipalities (97.9%) were successful in increasing at least one of the them: 151 (11.4%) achieved only one goal; 410 (31.1%) achieved at least two goals; and 731 (55.4%) achieved all goals. Importantly, 28 (2.1%) municipalities were not successful in any of the indicators. These data show that the total number of children under 10 with assessed nutritional status was 1,114,896 in 2022 (98.3% more compared to 2020); that 276,785 children had their food consumption markers registered in 2022 (a 236.7% increase compared to 2020); and that 17,675 individual visits of children with obesity under 10 were recorded in 2022 (a 262.9% increase compared to 2020) ^17^ ^,^ ^29^ ^,^ ^30^. More detailed monitoring will be presented elsewhere.

As discussed, the Brazilian Ministry of Health established a partnership with the UFAL to evaluate PROTEJA’s implementation and impact. Public policy implementation consists of efforts to apply government actions, including resource allocation and the development of planned processes. Implementation evaluation aims to assess whether the policy is implemented according to its planning, identifying whether inputs and processes are in line with expectations or can be improved. On the other hand, is the impact evaluation approach that allows to verify whether the policy is generating the expected results and impacts ^56^. Impact assessment will be conducted at the end of the first cycle (2021-2023).

## Final considerations

Despite investments in obesity prevention policies by many countries, which offer diffusion and evaluation potentials to researchers and governments, few are the experiences that have achieved some success. As such, countries should prioritize the creation and implementation of innovative programs and actions to reverse childhood obesity.

PROTEJA is an effort by the Brazilian Government to intensify the implementation of multiple local interventions for prevention and care of childhood obesity. No intervention alone can completely reverse the current scenario, but combining multiple interventions that tackle the multiple determinants of obesity, and fostering environments and municipalities that promote adequate and healthy eating and exercise is a good path.

Finally, despite implementations challenges, the Brazilian proposal is innovative and presents a possible path and an international example to contribute to childhood obesity reversal, besides adding to implementation sciences.
